# Development of venous thromboembolism and its impact on hospitalized adults with covid-19: rapid systematic review

**DOI:** 10.1590/1677-5449.202400732

**Published:** 2025-03-14

**Authors:** Andressa Pereira Rocha, João Gabriel Sanchez

**Affiliations:** 1 Hcor, São Paulo, SP, Brasil.; 2 Universidade de Brasília – UnB, Brasília, DF, Brasil.

**Keywords:** covid-19, SARS-CoV-2, thrombosis, venous thromboembolism, deep vein thrombosis, pulmonary embolism

## Abstract

The association between COVID-19 and coagulation disorders has been discussed since the onset of the pandemic. Four years into the pandemic, it is crucial to organize the findings and evidence accumulated thus far. The objective of this study was to review and synthesize the available scientific evidence regarding the relationship between COVID-19 and development of venous thromboembolism (VTE). A rapid systematic review was conducted by searching two electronic databases, selecting systematic review articles that assessed the association between COVID-19 and development of VTE, such as deep vein thrombosis (DVT) or pulmonary embolism (PE). The studies indicated that hospitalized COVID-19 patients are at greater risk of developing VTE, especially those admitted to intensive care units (ICUs). Elevated D-dimer levels and male gender were also associated with increased risks.

## INTRODUCTION

The COVID-19 outbreak was declared a pandemic by the World Health Organization (WHO) on March 11 of 2020 and studies from that year were already discussing the coagulation disorders, specifically hypercoagulation, observed in patients admitted because of the disease.^1^ In addition to the most common clinical findings (fever, coughing, and fatigue), these studies also describe changes observed in laboratory test results (hemostatic markers such as D dimer, fibrinogen, and activated partial thromboplastin time, for example) and report increased lethality rates, observing occurrence of thrombotic events such as disseminated intravascular coagulation, deep venous thrombosis, and pulmonary thromboembolism.^1,2^

When discussing coagulation, one should consider Virchow’s triad, which associates thrombosis with three risk factors: endothelial injury, blood stasis, and hypercoagulability.^3^ Coagulation is activated by both intrinsic and extrinsic factors and the physiology of coagulation is regulated by three anticoagulant mechanisms: the antithrombin system, the C protein activation system, and tissue factor pathway inhibitor.^4^

Systemic infection can provoke dysfunction of the coagulation mechanisms and, in conjunction with the immune response it provokes, can trigger endothelial events that impact hemostasis,^5^ such as endothelial damage, which is one of the factors that make up Virchow’s triad.^4^

Scientific publications identify pulmonary thromboembolism as one of the potential complications of COVID-19, both because of elevation of inflammatory cytokine levels and because of tachyarrhythmias and bradyarrhythmia.^1,2,6^ A thrombotic event such as pulmonary embolism can cause significant hemodynamic instability, leading to severe clinical outcomes, such as cardiorespiratory arrest, if not treated adequately.^7,8^ Additionally, although less frequent, cases of arterial thrombosis have also been observed and contribute to the morbidity and mortality associated with COVID-19, worsening these patients’ prognosis even further.^9^

In view of this, it is important to understand and monitor the advances in understanding achieved through research over the last 4 years with regard to the vascular effects of an infection such as COVID-19, contributing to evidence-based practice, both in respect of this disease specifically and also of the body’s response to new pathogens in general. As such, the objective of this review is to identify and synthesize the scientific evidence contained in studies that have assessed the association between COVID-19 and development of venous thromboembolism (VTE) as one of the possible outcomes of the coagulation disorders it provokes.

## METHODS

A rapid systematic review was conducted of the literature published up to December 2023 to map studies that have assessed the association between COVID-19 and development of thromboembolic events such as pulmonary embolism (PE) and deep venous thrombosis (DVT), using the Cochrane methodology for systematic reviews,^10^ adapted for a rapid study design.^11^ Results are reported as set out in the 2020 Preferred Reporting Items for Systematic Reviews and Meta-Analyses (PRISMA).^12^

A rapid review is a type of systematic review that is conducted within a very short time frame, taking methodological shortcuts that enable conclusions to be drawn sooner, while having minimal impact on study quality.^11^ The shortcuts chosen for the review presented herein and their possible limitations are described in Table 1.

**Table 1:** Shortcuts taken and their expected impacts on this review as limiting factors of the rapid review methodology.

Review stage	Shortcut taken	Potential impact on the study*
Data extraction	Data extracted by one investigator only	Increased number of errors. However, the impact on the results is unknown.
Literature search	Searches limited to two databases.	No impairment as long as at least two databases are used.
Inclusion criterion	Grey literature omitted.	Introduces publication bias (reduces AMSTAR2** assessment scores by 1 point).
Synthesis and analysis of data	No meta-analysis conducted; synthesis produced by just one author.	Impact unknown. Meta-analysis can increase the power and precision of the analysis. However, if conducted incorrectly, it has the potential to generate misleading results. No points are deducted in AMSTAR2 assessments if this step is eliminated because of heterogeneous studies.

* The potential impacts are described as per Haby et al.;^11^

** AMSTAR2 = Assessing the Methodological Quality of Systematic Reviews (tool for assessing the methodological quality of literature reviews).^13^

This review is intended to answer the following question: what impact has COVID-19 had on the number of cases of venous thromboembolism among hospitalized adults? This question was formulated using the PECO acronym, as follows, P (population): hospitalized adults; E (exposure): COVID-19; C (comparison): unspecified; and O (outcome): venous thromboembolism cases.

The review protocol was developed and published before starting the bibliographic searches and is registered and available on the Zenodo platform.^14^

### Eligibility criteria

Studies were selected according to the following inclusion criteria: articles describing systematic reviews of studies that investigated the association between COVID-19 and development of VTE such as DVT and PE in adult hospitalized patients, published in English, Spanish, or Portuguese since 2020, when the pandemic began.

Exclusion criteria were publications that were not original scientific articles of primary case studies and non-systematic reviews. Studies were also excluded if they covered COVID-19 or coagulation disorders, but did not assess their association, or were studies of coagulopathy in general (without focusing on VTE) or post mortem analyses.

### Search strategy and study selection

Searches were run on the PubMed and Biblioteca Virtual em Saúde (BVS) electronic databases. Initially, a search string was constructed for PubMed and then it was adapted for BVS. The strings were constructed using each database’s controlled vocabulary, synonyms, and keywords combined using Boolean operators, to include the groups of terms defined by the PECO question. The full search strings for each database are presented in the Supplementary material.

The Rayyan platform was used to manage the results of the database searches,^15^ for both stages of study selection: 1) screening by reading titles and abstracts; and 2) confirmation of eligibility by reading full texts. Both stages were conducted independently by two reviewers who reached consensus through dialogue in cases of disagreement.

### Data extraction

Data were extracted from the studies selected by one reviewer and were then checked by the other using a spreadsheet prepared in advance.

According to the 2022 Thromboembolism Guidelines,^3^ the outcome of interest (blood coagulation disorders, due to a hypercoagulable state in the case of VTE) can be assessed using several methods, preferably combined with each other: clinical assessment, imaging exams (vascular ultrasonography with Doppler), application of the Wells et al. score,^16^ and/or serum D dimer (a product of fibrin degradation). Therefore, for studies that described a method of assessing blood coagulability, this was extracted and duly registered in a table. It was not therefore necessary to exclude any studies purely because of the measure used.

### Differences between the original protocol and the final study

As the study progressed, certain changes were made to the protocol to improve its findings: 1) the inclusion and exclusion criteria were changed to include systematic reviews exclusively; 2) the outcomes of interest (coagulation disorders), which can be diverse, were restricted to VTE only, encompassing DVT and PE.

### Methodological quality assessment

The methodological quality of reviews was assessed using the AMSTAR2 tool (Assessing the Methodological Quality of Systematic Reviews).^13^ This analysis was conducted by one reviewer and validated by another. This tool assesses the methodological quality of the systematic reviews included in the article, identifying domains that can introduce research biases and affect the reporting of review results.

## RESULTS

**Figure 1:**
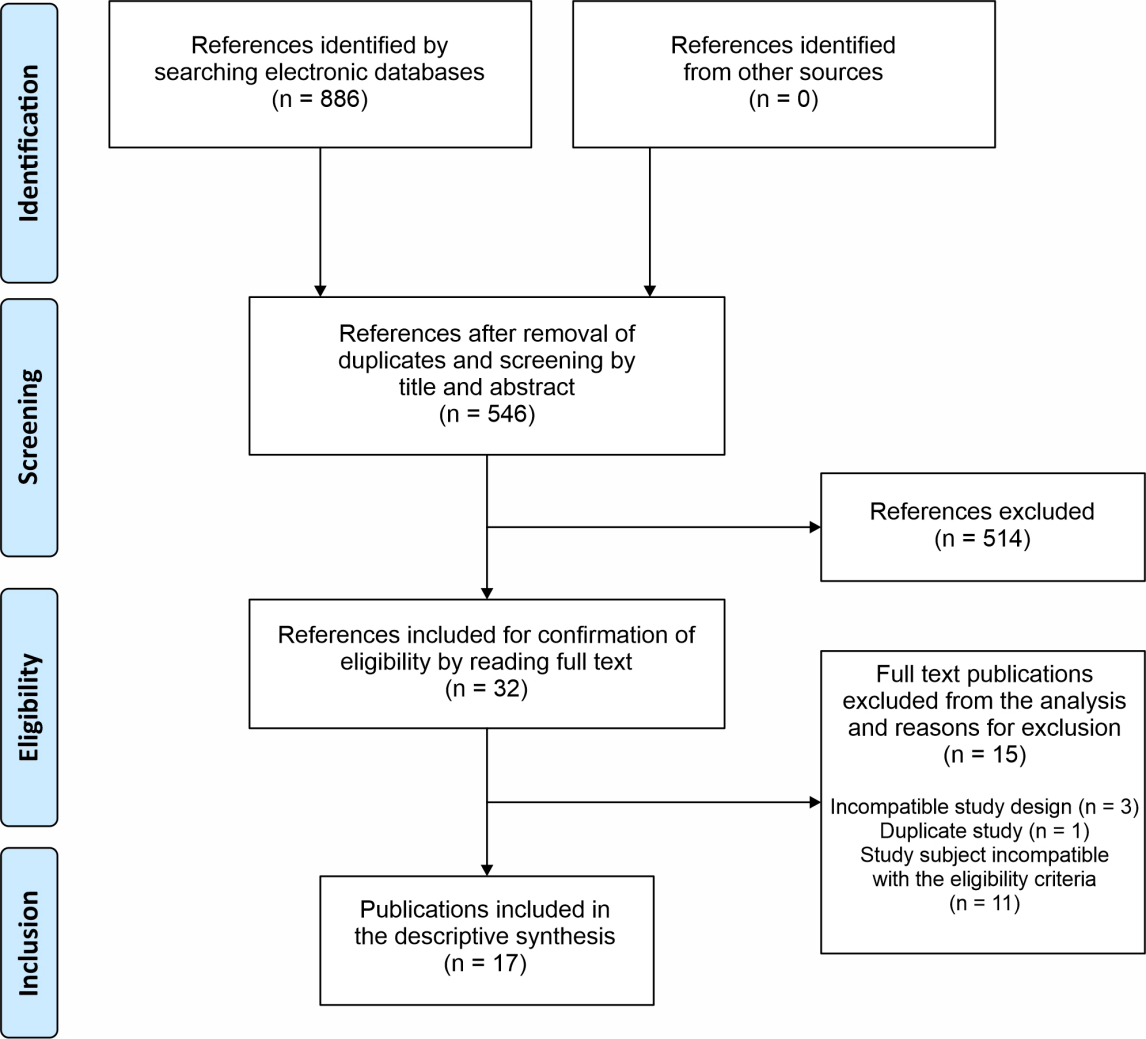
Review flow diagram, illustrating the numbers of studies identified, included, and excluded in each stage, according to the PRISMA 2020 model.^12^

A total of 886 publications were identified initially and 17 articles were included in the review for analysis and interpretation of the results after the process of selection based on the eligibility criteria.^17-33^Figure 1 presents a flow diagram illustrating the steps involved in study selection.

Thirteen (76.47%) of the 17 systematic reviews conducted meta-analyses. The incidence of VTE was analyzed in 10 reviews (58.82%), while six (35.29%) investigated the prevalence of VTE, and one (5.88%) assessed the pathophysiology of VTE in relation to COVID-19 infection. Overall, 15 studies dealt with both DVT and PE, one only covered DVT, and one only analyzed PE. The study population comprised hospitalized adult patients in all studies, either in ICUs, in wards, or in both. Table 2 lists the main characteristics of the studies included in the review.

**Table 2:** Characteristics of the studies selected for data extraction.

Authors	Type of study	Types of studies included	Number of studies included	Date of last literature search	Results	Conclusions	AMSTAR2 score^12^
Mota et al.^17^	Systematic review	Cohort	6	March/2021	VTE and PE rates: One study observed that 34% of cases of VTE were identified at hospital admission and that patients who needed intensive care had higher VTE rates in both of the groups observed. Additionally, it was observed that 4.4% of the patients studied exhibited thromboembolic complications; 63% of them developed PE and 50% had VTE.	Patients with COVID-19 demonstrated a greater tendency to develop VTE and PE. In this infectious condition, in common with others, there is a generalized manifestation of inflammation and one of the main laboratory markers is increased D dimer levels, which are correlated with the severity of clinical symptoms.	Critically low
Maitra et al.^18^	Systematic review and meta-analysis	Cohort and RCTs	19	July/2020	Incidence of VTE: - Incidence of VTE: 23% (10-36%; 95%CI) - Incidence of PE: 12% (6-17%; 95%CI) - Incidence of DVT: 15% (8-23%; 95%CI)	It was observed that prevalence of PE and DVT was elevated among individuals with COVID-19 and that thromboembolic events in general exhibited considerable heterogeneity. This variation could be attributable to factors such as the ethnic diversity of patients, differences between screening and prophylaxis protocols, exacerbation of clinical status, and presence of comorbidities.	Critically low
Henrina et al.^19^	Systematic review and meta-analysis	Retrospective, prospective, and cross-sectional cohort	45	November/2020	Association between VTE and several different factors: There were significant associations between VTE and factors such as admission to an ICU (1.53-3.52; 95%CI), male sex (1.08-1.35; 95%CI), elevated D dimer (2.74-6.25; 95%CI), LDH (19.33-122.54; 95%CI), and advanced age (0.06-5.53; 95%CI).	Occurrence of VTE among patients affected by COVID-19 is directly related to a greater probability of admission to an ICU. Moreover, factors such as male sex, advanced age, and elevated white cell counts and D dimer levels are associated with a greater risk of development of VTE during the COVID-19 infection.	Critically low
Suh et al.^20^	Systematic review and meta-analysis	Prospective and retrospective cohort	27	June/2020	Incidence of PE and DVT: - Incidence of PE: 16.5% (95%CI: I2 = 0.93) - Incidence of DVT: 14.8% (95%CI: I2 = 0.94) PE was found more frequently among patients admitted to the ICU (24.7% [95%CI]) than in those not admitted to the ICU (10.5% [95%CI]). DVT was present in 42.4% of the patients with PE.	More than half of the patients who developed PE did not present signs of concomitant DVT. The D dimer cutoffs used as criterion to rule out occurrence of PE, as defined in preexisting guidelines, appear to also be applicable to patients diagnosed with COVID-19.	Low
Kollias et al.^21^	Systematic review and meta-analysis	Observational	47	September/2020	Prevalence of PE and DVT: - Prevalence of PE: 32% (25-40%; 95%CI) - Prevalence of DVT: 27% (21-34%; 95%CI)	In patients hospitalized with COVID-19 and screened or assessed for VTE, the prevalence of DVT and PE are each approximately 30%, even applying thromboprophylaxis measures. Notably, the risk of VTE appeared to be higher in comparison with patients who did not have COVID-19 who were admitted to the same ICUs.	Critically low
Mazzaccaro et al.^22^	Systematic review	Randomized clinical trials, cohort studies, and cases series	69	May/2021	Prevalence of VTE: - Prevalence of VTE: 16.7% (5.8-30%) and higher among patients in the ICU (60.8-85.4%). Advanced age, elevated D dimer, and obesity increased the chances of developing VTE. Female sex appeared to be protective against VTE.	VTE occurrence was frequently observed among patients hospitalized for COVID-19, with particularly elevated prevalence rates among patients admitted to the ICU. Factors such as advanced age, obesity, and elevated D dimer levels at admission were associated with an increased probability of VTE, whereas female sex was demonstrated as being protective against development of VTE.	Critically low
Gabbai-Armelin et al.^23^	Systematic review and meta-analysis	Cross-sectional and case-control cohort.	20	Information not provided	Prognosis linked to: Hypertension and diabetes were the comorbidities most frequently associated with thrombotic events.	Individuals over the age of 60 years who had a history of hypertension, diabetes, and D dimer concentrations exceeding 3.17 μg/mL were identified as prognostic factors associated with development of COVID-19-related VTE.	Low
Roncon et al.^24^	Systematic review and meta-analysis	Retrospective and prospective cohort	23	August/2020	Incidence of PE: - PE incidence: 14.7% (9.9-21.3%; 95%CI; I2 = 95.0%) for patients in wards and 23.4% (16.7-31.8%; 95%CI; I2 = 88.7%) for patients in an ICU.	Occurrence of PE in a hospital setting is more frequent in patients with COVID-19 who are in intensive care than among those who are in general wards. There are also indications that the number of cases of PE may be underestimated.	Critically low
Xiong et al.^25^	Systematic review and meta-analysis	Retrospective, prospective, and case-control cohort	12	July/2020	Prevalence of thrombosis: - Prevalence of thrombosis: 22% (0.08-0.40; 95%CI) in patients in wards and 43% (0.29-0.65; 95%CI) in patients in ICUs. Compared with non-thrombotic patients, thrombotic patients exhibited higher D dimer levels. Age, platelet counts, and male sex tend to be risks.	There was a considerable prevalence of thrombosis in patients with COVID-19, especially those in ICUs, despite administration of pharmacological prophylaxis against VTE. It is therefore crucial to pay special attention to elevated D dimer levels, LDH, and leukocytes and to drops in lymphocyte levels in patients with COVID-19.	Critically low
Longchamp et al.^26^	Systematic review and meta-analysis	Retrospective and prospective cohort	33	June/2020	Incidence of VTE and PE: - Incidence of VTE: 9% (5-13%; 95%CI) in general; 21% (14-28%; 95%CI) for patients in an ICU. - Incidence of PE: 8% (4-13%; 95%CI) in general; 17% (11-25%; 95%CI) for patients in an ICU. - Incidence of DVT: 3% (1-5%; 95%CI) in general; 8% (3-14%; 95%CI) for patients in an ICU.	There is considerable potential for severe thromboembolic events in patients hospitalized with COVID-19. However, incidence is sensitive to disease severity. These findings confirm the need to implement thromboprophylactic measures in all patients hospitalized for COVID-19, in addition to conducting clinical trials that assess different thromboprophylaxis protocols in subpopulations.	Low
Nopp et al.^27^	Systematic review and meta-analysis	Cross-sectional and case-control cohort,	86	August/2020	Prevalence of VTE and PE: - Prevalence of VTE: 7.9% (5.1-11.2; 95%CI) in patients not in an ICU and 22.7% (18.1-27.6; 95%CI) for patients in an ICU. - Prevalence of PE: 3.5% (2.2-5.1; 95%CI) in patients not in an ICU and 13.7% (10.0-17.9; 95%CI) for patients in an ICU.	There is an elevated risk of VTE in the ICU. However, it is also high in hospitalized patients not in an ICU setting. Notably, patients who had VTE had significantly higher D dimer levels.	Low
Jenner et al.^28^	Systematic review	Observational studies	28	November/2020	Rate of thrombotic complications: Thrombotic complications occurred in 34% of the patients treated in the ICU, with DVT reported in 16.1% and PE in 12.6%, despite anticoagulant thromboprophylaxis.	Even after administration of anticoagulant thromboprophylaxis, a considerable incidence of thrombotic complications was observed in patients with COVID-19 receiving intensive care in an ICU. Additionally, implementation of systematic screening reveals a significant number de thrombotic complications that may not be investigated on the basis of clinical suspicion alone. Systematic screening for VTE is strongly recommended.	Low
Wu et al.^29^	Systematic review and meta-analysis	Prospective and retrospective studies	39	September/2020	Incidence of PE and VTE: Incidence of PE: 17% (13-21; 95%CI) Incidence of VTE: 42% (25-60; 95%CI) Male patients with COVID-19 have a higher probability of VTE.	VTE is frequently observed as a complication in patients severely affected by COVID-19 and is robustly related to adverse clinical outcomes.	Low
Birkeland et al.^30^	Systematic review	Retrospective, prospective, and cross-sectional cohort	14	June/2020	Incidence of VTE: Incidence of VTE: 26.9% (20.8-33.1; 95%CI). The likelihood of VTE was greater in the ICU (OR = 6.38; 3.67-11.11; 95%CI).	Even with prior anticoagulation, the incidence of VTE was considerably elevated.	Critically low
Mansory et al.^31^	Systematic review and meta-analysis	Retrospective cohort, prospective cohort, cross-sectional, RCTs, and case series	91	December/2020	VTE epidemiology: The overall frequencies of VTE in ICU and non-ICU patients were 24.1% (20.07-28.28; 95%CI) and 7.7% (20.07-28.28; 95%CI) respectively. PE occurred in 8.5% (6.91-10.20; 95%CI) and proximal DVT occurred in 8.2% (6.67-9.87; 95%CI) of all hospitalized patients.	There is a high incidence of VTE in patients hospitalized with COVID-19, especially those in the ICU. However, sensitivity analyses indicate that previously reported rates of VTE in COVID-19 may have been overestimated.	Critically low
Porfidia et al.^32^	Systematic review and meta-analysis	Cohort	30	June/2020	Incidence of VTE: - Incidence of VTE: 24% (95% PI, 5-66%) in patients in the ICU and 9% (PI 95%, 0-94%) in patients in wards. - Incidence of PE: 19% (95% PI, 6%-47%) in patients in the ICU and 4% (PI 95%, 0-100%) in patients in wards. -Incidence of DVT: 7% (95% PI, 0-69%) in patients in the ICU and 7% (95%CI, 1-49%) in patients in wards.	VTE is a common complication in patients admitted for COVID-19 and PE is a recurrent presentation. It is crucial to maintain a high level of clinical suspicion in order to identify cases promptly.	Critically low
Zhang et al.^33^	Systematic review and meta-analysis	Prospective and retrospective studies	40	August/2020	Prevalence of VTE: - Prevalence of VTE: 7% in patients not in the ICU (0.01-0.18; 95%CI) and 31% in patients in the ICU (0.22-0.42; 95%CI). ICU patients also had a higher prevalence of PE, 17% (0.12-0.23; 95%CI), and a greater prevalence of DVT, 25% (0.14-0.37; 95%CI).	VTE is a frequent occurrence in patients hospitalized with COVID-19, particularly among those in the ICU. Implementation of screening tests for PE and DVT could significantly improve detection rates in patients with COVID-19, both in the ICU and in the wards, compared with tests based exclusively on clinical suspicion.	Low

AMSTAR2 = Assessing the Methodological Quality of Systematic Reviews;^12^ RCTs = randomized clinical trials; PE = pulmonary embolism; I2 = heterogeneity index; PI = prediction interval; LDH = lactate dehydrogenase; OR = odds ratio; VTE = venous thromboembolism; DVT = deep vein thrombosis; ICU = Intensive care unit.

With regard to D dimer, 12 studies (70.58%) identified an association between elevated levels of this marker and risk of development of VTE in COVID-19 cases, showing that patients with VTE had higher levels.^17,19-23,25,27,29,30^

Moreover, five studies (29.4%) showed that males were at greater risk of VTE as a complication of COVID-19.^18,19,22,25,29^

Additionally, three studies (17.64%) suggested that systematic screening using imaging exams to investigate VTE is strongly recommended because it can identify complications that may go unnoticed with clinical assessment only.^28,32,34^

Finally, 15 studies (88.23%) demonstrated that the risk of development of VTE is higher among patients in an ICU than among hospitalized patients who are not in an ICU, although these patients are also at elevated risk.^17,19-22,24-32,34^

## DISCUSSION

Of the known coagulation disorders, the inflammatory response in COVID-19 primarily manifests with a hypercoagulable state, one of the outcomes of which is VTE.^5^ In view of this, the present study focused on this outcome, studying PE and DVT specifically.

COVID-19 is the infectious disease caused by the SARS-CoV-2 virus and responsible for the 2020 to 2023 pandemic. It had a worldwide impact on public health and many questions about its pathophysiology and repercussions have not yet been answered. It is known that the disease predominantly causes respiratory manifestations with secondary cardiovascular manifestations, such as coagulopathy. Moreover, increasing numbers of studies have proven its robust relationship with thromboembolic events caused by coagulation disorders triggered by inflammatory response mechanisms.^34^

The pathophysiology of COVID-19 involves a series of complex events that occur in the body in response to infection by the virus, such as the inflammatory response, which involves release of proinflammatory cytokines including interleukin-6 (IL-6). In some cases, this inflammatory response may be exacerbated, leading to a condition known as a “cytokine storm”, which is associated with serious complications.^35^ The virus can also injure the endothelial cells that line blood vessels, contributing to coagulation system activation and possibly resulting in vascular thrombosis. These events induce the hypercoagulable state frequently observed in patients with COVID-19.^36^

This review identified evidence from other reviews that identify VTE as a condition that occurs in a significant proportion of patients hospitalized with COVID-19. This manifestation is clearly more common among patients in ICUs, which can be explained by the fact that this type of patient meets all three criteria in Virchow’s triad, in addition to very often being subjected to immobility for prolonged periods. Notwithstanding, it was also possible to observe in the findings of the studies reviewed that patients who are not admitted to an ICU are also exposed to risks.

It is pertinent to point out that collecting data during a pandemic involves major challenges. In the case of the COVID-19 pandemic, the emergent nature and rapid propagation of the outbreak meant it was necessary to generate information rapidly in order to guide clinical decision-making, which generated an accelerated and productive scientific research environment. However, the extensive limitations prevailing at the time, such as sanitary restrictions, lack of resources, and different management approaches, all affected the ability to conduct clinical studies, resulting in greater dependence on secondary data. As a result, much of the data available during the pandemic were obtained from observational studies and hospital records, which, while valuable, can introduce biases and limit the scope for interpretation of results.

### Study limitations

The findings of this review confirm the current literature with regard to the subject studied. However, there are still many gaps in knowledge about the subject and about the pathophysiology of COVID-19 itself. We acknowledge that adoption of the methodological shortcut of restricting searches to just two databases, could have contributed to these gaps, although, as described in Table 1, this is not expected to be the case. Although there are many primary studies of the subject, they may not have been included in reviews of the literature.

It is also pertinent that none of the reviews included and analyzed were rated as having high methodological quality according to the AMSTAR2 tool.^13^ Therefore, the results and the considerations presented should be used with care, since the low or critically low methodological quality ratings of these reviews indicate possible biases in their descriptions of findings.

This review did not investigate mortality or the possible treatments for the conditions analyzed, exclusively focusing on the intrahospital results. Future research could investigate the long-term effects and management of COVID-19 associated with VTE. Another relevant limitation of this study is that the majority of the articles included are from 2020 and 2021. We reiterate that one inclusion criterion for this review was to only include systematic reviews, which, on one hand, does not indicate a lack of primary studies, but, on the other, does highlight the need to conduct reviews of the literature on the subject, in order to furnish more syntheses of the available scientific evidence.

### Contributions to clinical practice

Based on our findings, it is suggested that reviews be conducted to investigate the comparison “ICU patient without COVID-19 vs. ICU patient with COVID-19”, in order to refine the data. It is also suggested that an instrument for VTE risk assessment be developed specifically for patients with COVID-19 in ICUs.

It is essential to continue investigating the subject and publishing the findings in order to ensure that the subject continues contributing to society and to health professionals interested in it.

## CONCLUSIONS

Working from the available scientific evidence, and considering the scant availability of secondary studies and the low methodological quality of those that do exist, we identified and synthesized three main points regarding the association between COVID-19 infections and development of VTE. First, there is an elevated incidence of thromboembolic events (DVT and/or PE) emerging as complications associated with COVID-19 among hospitalized adults, since this condition triggers a systemic inflammatory response in the body, which can frequently cause coagulation disorders, such as hypercoagulability and endothelial injury, especially among patients in ICUs who are exposed to additional risk factors such as immobility and endothelial injury secondary to use of vascular devices. Second, elevated D dimer levels are often found in the patients with the most severe cases and this is an indicator of poor prognosis because it is associated with a higher risk of thromboembolic complications. Third, it is necessary to be more vigilant, screening for VTE in hospitalized patients with COVID-19 using laboratory markers such as D dimer and imaging exams, and, where appropriate, providing antithrombotic prophylaxis as recommended in the guidelines, in order to avert complications.

## Supplementary Material

Supplementary material accompanies this paper.

Supplementary table 1. Database, search strategy, and number of publications identified.

Supplementary table 2. List of articles excluded during selection of data and the reasons or exclusion.

This material is available as part of the online article from https://doi.org/10.1590/1677-5449.202400732
