# Supplementary material for: Development of venous thromboembolism and its impact on hospitalized adults with covid-19: rapid systematic review
Source: J Vasc Bras. 2025 Mar 14;24:e20240073. doi: 10.1590/1677-5449.202400732 (PMC11924586; doi:10.1590/1677-5449.202400732)
Supplement: Supplementary table 1. [file jvb-24-e20240073-suppl01.pdf]

Supplementary table 1. Database, search strategy, and number of publications identified.

**Database**	**Search strategy**	**Results**
PubMed	((“Blood Coagulation Disorders”[MeSH Terms] OR (“Coagulation”[Title/Abstract] OR “Blood Coagulation Disorders”[Title/Abstract] OR “Thrombosis”[Title/Abstract] OR “Thromboembolism”[Title/Abstract] OR “pulmonary embolism”[Title/Abstract])) AND (“COVID-19”[MeSH Terms] OR “sars cov 2”[MeSH Terms] OR (“COVID-19”[Title] OR “sars cov 2”[Title]))) AND ((systematicreview[Filter]) AND (2020:3000/12/12[pdat]) AND (english[Filter] OR portuguese[Filter] OR spanish[Filter]))	337
BVS	((mh:(COVID-19)) OR (ti:(COVID-19)) OR (ti:(SARS-COV-2))) AND ((coagulation) OR (“pulmonary embolism”) OR (thrombosis) OR (thromboembolism) OR (“blood coagulation disorders”) OR (mh:(“Blood Coagulation”)) OR (embolismo pulmonar) OR (“Coagulação Sanguínea”) OR (“Coagulación Sanguínea”))	549

BVS = Biblioteca Virtual em Saúde

Supplementary table 2. List of articles excluded during selection of data and the reasons or exclusion.

**Citation**	**Article title**	**Reason for exclusion**
Sarkar et al., 2021	COVID-19 and coagulopathy	Not a systematic review
Lippi et al., 2021	Coronavirus disease 2019 associated coagulopathy	Not a systematic review
Harrison et al., 2021	Cardiovascular risk factors, cardiovascular disease, and COVID-19: an umbrella review of systematic reviews	Not a systematic review
Xiaolin et al., 2020	Coagulopathy in patients with coronavirus disease 2019 (COVID-19): a systematic review and meta-analysis	Coagulopathy in general
Lin et al., 2020	COVID-19 and coagulation dysfunction in adults: a systematic review and meta-analysis	Coagulopathy in general
Xiao et al., 2022	Cumulative evidence for the association of thrombosis and the prognosis of COVID-19: systematic review and meta-analysis	Divergent outcome

Citation	Article title	Reason for exclusion
Castillo-Perez et al., 2021	Differences between surviving and non-surviving venous thromboembolism COVID-19 patients: a systematic review	Postmortem analysis
X. Zhou et al., 2021	Incidence and impact of disseminated intravascular coagulation in COVID-19 a systematic review and meta-analysis	Coagulopathy in general
Young et al.[19]	Pulmonary embolism and deep vein thrombosis in COVID-19: a systematic review and meta-analysis	Duplicated
Overton et al., 2022	Pulmonary thromboembolic events in COVID-19: a systematic literature review	Postmortem analysis
Massoud et al., 2023	Risk of thromboembolic events in non-hospitalized COVID-19 patients: a systematic review	Patients not in hospital
Mitra et al., 2021	Severe COVID-19 and coagulopathy: a systematic review and meta-analysis	Coagulopathy in general
Uapraset et al., 2020	Systemic coagulopathy in hospitalized patients with coronavirus disease 2019: a systematic review and meta-analysis	Coagulopathy in general

**Citation**	**Article title**	**Reason for exclusion**
Pierre et al., 2021	Venous thromboembolism in COVID-19: systematic review of reported risks and current guidelines	Focused on guidelines
Srisvatava et al., 2021	Venous thromboembolism is linked to severity of disease in COVID-19 patients: A systematic literature review and exploratory meta-analysis	Divergent outcome

Tabela suplementar 1. Base de dados, estratégia de busca e número de publicações encontradas.

**Base de dados**	**Estratégia de busca**	**Resultados**
PubMed	((“Blood Coagulation Disorders”[MeSH Terms] OR (“Coagulation”[Title/Abstract] OR “Blood Coagulation Disorders”[Title/Abstract] OR “Thrombosis”[Title/Abstract] OR “Thromboembolism”[Title/Abstract] OR “pulmonary embolism”[Title/Abstract])) AND (“COVID-19”[MeSH Terms] OR “sars cov 2”[MeSH Terms] OR (“COVID-19”[Title] OR “sars cov 2”[Title]))) AND ((systematicreview[Filter]) AND (2020:3000/12/12[pdat]) AND (english[Filter] OR portuguese[Filter] OR spanish[Filter]))	337
BVS	((mh:(COVID-19)) OR (ti:(COVID-19)) OR (ti:(SARS-COV-2))) AND ((coagulation) OR (“pulmonary embolism”) OR (thrombosis) OR (thromboembolism) OR (“blood coagulation disorders”) OR (mh:(“Blood Coagulation”)) OR (embolismo pulmonar) OR (“Coagulação Sanguínea”) OR (“Coagulación Sanguínea”))	549

BVS = Biblioteca Virtual da Saúde

Tabela suplementar 2. Lista de artigos excluídos durante a seleção de dados e sua respectiva justificativa.

**Citação**	**Título do artigo**	**Razão da exclusão**
Sarkar et al., 2021	Covid-19 and coagulopathy	Não é revisão sistemática
Lippi et al., 2021	Coronavirus disease 2019 associated coagulopathy	Não é revisão sistemática
Harrison et al., 2021	Cardiovascular risk factors, cardiovascular disease, and covid-19: an umbrella review of systematic reviews	Não é revisão sistemática
Xiaolin et al., 2020	Coagulopathy in patients with coronavirus disease 2019 (covid-19): a systematic review and meta-analysis	Coagulopatia em geral
Lin et al., 2020	Covid-19 and coagulation dysfunction in adults: a systematic review and meta-analysis	Coagulopatia em geral
Xiao et al., 2022	Cumulative evidence for the association of thrombosis and the prognosis of covid-19: systematic review and meta-analysis	Desfecho divergente

**Citação**	**Título do artigo**	**Razão da exclusão**
Castillo-Perez et al., 2021	Differences between surviving and non-surviving venous thromboembolism covid-19 patients: a systematic review	Análise *post mortem*
X. Zhou et al., 2021	Incidence and impact of disseminated intravascular coagulation in covid-19 a systematic review and meta-analysis	Coagulopatia em geral
Young et al.[19]	Pulmonary embolism and deep vein thrombosis in covid-19: a systematic review and meta-analysis	Duplicata
Overton et al., 2022	Pulmonary thromboembolic events in covid-19: a systematic literature review	Análise *post mortem*
Massoud et al., 2023	Risk of thromboembolic events in non-hospitalized covid-19 patients: a systematic review	Pacientes não hospitalizados
Mitra et al., 2021	Severe covid-19 and coagulopathy: a systematic review and meta-analysis	Coagulopatia em geral
Uapraset et al., 2020	Systemic coagulopathy in hospitalized patients with coronavirus disease 2019: a systematic review and meta-analysis	Coagulopatia em geral

**Citação**	**Título do artigo**	**Razão da exclusão**
Pierre et al., 2021	Venous thromboembolism in covid-19: systematic review of reported risks and current guidelines	Foco em diretrizes
Srisvatava et al., 2021	Venous thromboembolism is linked to severity of disease in covid-19 patients: A systematic literature review and exploratory meta-analysis	Desfecho divergente
