# Supplementary material for: Development of venous thromboembolism and its impact on hospitalized adults with covid-19: rapid systematic review
Source: J Vasc Bras. 2025 Mar 14;24:e20240073. doi: 10.1590/1677-5449.202400732 (PMC11924586; doi:10.1590/1677-5449.202400732)
Supplement: Translation

# Translation: Desenvolvimento de tromboembolismo venoso e seu impacto em adultos hospitalizados com covid-19: revisão sistemática rápida

**DOI:** 10.1590/1677-5449.202400731

**Authors:** Andressa Pereira Rocha, João Gabriel Sanchez

**Affiliations:** 1 Hcor, São Paulo, SP, Brasil.; 2 Universidade de Brasília – UnB, Brasília, DF, Brasil.

**Keywords:** covid-19, SARS-CoV-2, trombose, tromboembolismo venoso, trombose venosa profunda, embolia pulmonar

## Abstract

A associação entre a covid-19 e distúrbios de coagulação é discutida desde o início da pandemia, de modo que, passados 4 anos, é importante sistematizar as descobertas e evidências encontradas até o momento. O objetivo do estudo foi revisar e sintetizar as evidências científicas disponíveis sobre a relação entre a covid-19 e o desenvolvimento de tromboembolismo venoso. Foi realizada uma revisão sistemática rápida, com a busca conduzida em duas bases de dados eletrônicas, e incluídos artigos de revisão sistemática que avaliaram a associação entre a covid-19 e o desenvolvimento de tromboembolismo venoso, como trombose venosa profunda ou embolia pulmonar. Os estudos apontaram que pacientes hospitalizados por covid-19 apresentam maior risco de desenvolver tromboembolismo venoso, especialmente os internados em unidade de terapia intensiva. Valores de Dímero D elevado e o sexo masculino também foram associados a maiores riscos.

## INTRODUÇÃO

Estudos datados de 2020, início da pandemia de covid-19 – declarada pela Organização Mundial da Saúde (OMS) em 11 de março daquele ano –, já discutiam sobre distúrbios de coagulação, especificamente a hipercoagulação, observada em pacientes internados em razão da doença^1^. Além dos achados clínicos mais comuns (febre, tosse e fadiga), essas pesquisas descrevem alterações em exames laboratoriais (marcadores hemostáticos como Dímero D, fibrinogênio e tempo de tromboplastina parcial ativada, por exemplo) e aumento nos índices de letalidade, observando-se a ocorrência de eventos trombóticos como a coagulação intravascular disseminada, a trombose venosa profunda e o tromboembolismo pulmonar^1,2^.

Quando se fala em coagulação, deve-se levar em consideração a tríade de Virchow, que associa a trombose a três fatores de risco: lesão endotelial, estase sanguínea e hipercoagulabilidade^3^. A coagulação é ativada por fatores intrínsecos e extrínsecos, e a fisiologia da coagulação é regulada por três mecanismos anticoagulantes: sistema antitrombina, sistema ativador da proteína C e inibição da via do fator tecidual^4^.

Uma infecção sistêmica pode provocar disfunção nos mecanismos de coagulação e, em conjunto com a resposta imunológica gerada, desencadear eventos endoteliais que impactam a hemostasia^5^, como o dano endotelial, um dos fatores que compõem a tríade de Virchow^4^.

Publicações científicas apontam o tromboembolismo pulmonar como uma das potenciais complicações da covid-19, tanto por causa da elevação nos níveis de citocinas inflamatórias quanto em função de taquiarritmias e bradiarritmias^1,2,6^. Um evento trombótico como a embolia pulmonar pode ocasionar instabilidade hemodinâmica significativa, levando a desfechos clínicos graves, como a parada cardiorrespiratória, se não tratado adequadamente^7,8^. Além disso, embora menos frequentes, casos de trombose arterial também são observados e contribuem para a morbimortalidade associada à covid-19, agravando ainda mais o prognóstico desses pacientes^9^.

Em vista disso, é importante compreender e acompanhar os avanços nas pesquisas ao longo dos últimos 4 anos no que tange aos efeitos vasculares de uma infecção como a da covid-19, visando contribuir para a prática baseada em evidências, tanto sobre a doença em si quanto sobre as respostas do organismo a patógenos novos. Dessa forma, esta revisão teve como objetivo identificar e sintetizar as evidências científicas de estudos que avaliaram a associação entre a ocorrência de covid-19 e o desenvolvimento de tromboembolismo venoso (TEV) como um dos possíveis desfechos dos distúrbios de coagulação.

## MÉTODOS

Foi realizada uma revisão sistemática rápida até dezembro de 2023 para mapear estudos que tenham avaliado a associação entre a ocorrência de covid-19 e o desenvolvimento de eventos tromboembólicos como embolia pulmonar (EP) e trombose venosa profunda (TVP), seguindo a metodologia para revisões sistemáticas de Cochrane^10^, adaptada para um delineamento rápido^11^. Os resultados estão reportados conforme a ferramenta Preferred Reporting Items for Systematic Reviews and Meta-Analyses (PRISMA) 2020^12^.

A revisão rápida é um tipo de revisão sistemática desenvolvida em um espaço de tempo mais curto, adotando atalhos metodológicos que permitem maior agilidade na conclusão e, ao mesmo tempo, tendo impacto mínimo na qualidade da pesquisa^11^. Com relação aos atalhos escolhidos para a revisão que será apresentada, suas possíveis limitações estão descritas na Tabela 1.

**Tabela 1:** Atalhos escolhidos e seus impactos esperados nesta revisão como pontos limitantes da metodologia de revisão rápida.

Etapa da revisão	Atalho adotado	Potencial impacto na pesquisa*
Extração dos dados	Extração de dados realizada por um só revisor.	Aumentar o número de erros. Porém, o impacto nos resultados não é conhecido.
Busca na literatura	Limitar a busca a duas bases de dados.	Sem comprometimento, desde que incluídas ao menos duas bases de dados
Critério de inclusão	Omitir literatura cinzenta.	Introduzir viés de publicação (reduz 1 ponto em avaliações do AMSTAR2**).
Síntese e análise de dados	Não realizar metanálise; síntese elaborada por apenas um revisor.	Impacto desconhecido. A metanálise pode aumentar o poder e a precisão da análise. Porém, quando conduzida inadequadamente, tem o potencial de gerar resultados enganosos. Não há perda de pontos no AMSTAR2 se a etapa não ocorrer devido à heterogeneidade.

* Os potenciais impactos foram descritos de acordo com Haby et al.^11^;

** AMSTAR2 = Assessing the Methodological Quality of Systematic Reviews (ferramenta de avaliação de qualidade metodológica de revisões de literatura)^13^.

A revisão visou responder à seguinte pergunta: qual o impacto da covid-19 no número de casos de tromboembolismo venoso em indivíduos adultos hospitalizados? Essa pergunta foi elaborada com base no acrônimo PECO: P (população), indivíduos adultos hospitalizados; E (exposição), covid-19; C (comparador), não especificado; O (*outcome*/desfecho), casos de tromboembolismo venoso.

O protocolo da revisão foi elaborado e publicado antes do início da pesquisa bibliográfica e está registrado e disponível na plataforma Zenodo^14^.

### Critérios de elegibilidade

Para selecionar os estudos, foram definidos os seguintes critérios de inclusão: artigos de revisão sistemática de estudos que tenham investigado a associação entre ocorrência de covid-19 e desenvolvimento de TEV, como TVP e EP, em pacientes adultos e hospitalizados, publicados em inglês, espanhol ou português a partir de 2020, início da pandemia.

Já os critérios de exclusão foram: publicações que não fossem artigos científicos originais de estudos primários de estudos de caso e revisões não sistemáticas. Foram excluídos também estudos que tratam da covid-19 ou de distúrbios de coagulação, mas que não avaliaram sua associação, ou estudos que tratam da coagulopatia de forma ampla (sem foco em TEV) ou que tenham realizado análise *post mortem*.

### Estratégia de busca e seleção dos estudos

A busca foi realizada nas bases de dados eletrônicas PubMed e Biblioteca Virtual da Saúde (BVS). Inicialmente, foi elaborada uma estratégia de busca para a PubMed, posteriormente adequada à BVS. Para elaborar a busca, foram utilizados termos do vocabulário controlado de cada base, juntamente com sinônimos e palavras-chave combinados entre si com operadores booleanos, conforme os grupos de termos previamente descritos no acrônimo PECO. As estratégias completas por base podem ser conferidas no Material suplementar.

Os resultados das buscas nas bases de dados foram gerenciados pela ferramenta Rayyan^15^, utilizada para as duas etapas de seleção do estudo: 1) triagem por leitura de título e resumo; e 2) confirmação da elegibilidade a partir da leitura na íntegra dos estudos. Essas etapas foram realizadas de forma independente por uma dupla de revisores que dialogaram para chegar a um consenso nos casos de conflito.

### Extração de dados

Os dados dos estudos selecionados foram extraídos por um revisor, enquanto outro checava-os em uma planilha previamente elaborada.

Segundo a Diretriz de Tromboembolismo de 2022^3^, o desfecho de interesse (distúrbios na coagulação sanguínea, no caso do TEV, devido ao estado de hipercoagulabilidade) pode ser avaliado de diversas formas, preferencialmente combinadas entre si: avaliação clínica, exame de imagem (ultrassonografia vascular com Doppler), aplicação do escore de Wells et al.^16^ e/ou nível sérico de Dímero D (produto da degradação de fibrina). Sendo assim, dos estudos que relataram algum método de avaliação da coagulabilidade sanguínea, este foi extraído para ser devidamente registrado em tabela. Assim, não foi necessário excluir estudos apenas pela medida utilizada.

### Diferenças entre o protocolo inicial e o estudo final

Para aprimorar os achados ao longo da revisão, algumas mudanças foram feitas em relação ao protocolo: 1) mudanças nos critérios de inclusão e exclusão, optando-se por incluir apenas revisões sistemáticas; 2) restrição dos desfechos de interesse (distúrbios de coagulação), que podem ser diversos, para TEV apenas, abrangendo a TVP e a EP.

### Avaliação da qualidade metodológica

Para analisar a qualidade metodológica das revisões, foi utilizada a ferramenta Assessing the Methodological Quality of Systematic Reviews (AMSTAR2)^13^. A análise foi realizada por um revisor e validada por um segundo revisor. Essa ferramenta avalia a qualidade metodológica das revisões sistemáticas incluídas neste artigo, identificando os domínios que implicam a ocorrência de vieses de pesquisa e relatando os resultados dessas revisões.

## RESULTADOS

Foram identificadas inicialmente 886 publicações e, após a etapa de seleção com base nos critérios de elegibilidade, 17 artigos foram incluídos no estudo para análise e interpretação dos resultados^17-33^. As etapas de seleção dos estudos são apresentadas no fluxograma da Figura 1.

**Figura 1:** Fluxograma da revisão com número de estudos identificados, incluídos e excluídos por etapa, conforme modelo PRISMA 2020^12^.

Entre as 17 revisões sistemáticas, 13 (76,47%) apresentaram metanálise. A incidência de TEV foi avaliada em 10 revisões (58,82%), enquanto seis (35,29%) investigaram a prevalência de TEV e uma (5,88%) a fisiopatologia do TEV em relação à infecção por covid-19. No total, 15 estudos abordaram TVP e EP, enquanto um abordou apenas TVP e um outro apenas EP. A população de todos os estudos compreendeu pacientes adultos e hospitalizados, em leito de UTI, de enfermaria ou em ambos. As principais características dos estudos incluídos estão descritas na Tabela 2.

**Tabela 2:** Características dos estudos incluídos após extração de dados.

Autor	Tipo de estudo	Tipos de estudos incluídos	Número de estudos incluídos	Data da última busca na literatura	Resultados	Conclusão	Pontuação AMSTAR2^12^
Mota et al.^17^	Revisão sistemática	Coorte	6	Março/2021	Taxas de TEV e EP: Um estudo observou que 34% dos casos de TEV foram identificados na admissão hospitalar e que pacientes de ambos os grupos observados que necessitaram de cuidados intensivos desenvolveram taxas mais elevadas de TEV. Além disso, observou-se que 4,4% dos pacientes estudados apresentaram complicações tromboembólicas; 63% deles desenvolveram EP e 50% TEV.	Pacientes com covid-19 demonstraram maior tendência a desenvolver TEV e EP. Nesse quadro infeccioso, assim como em outros, há uma manifestação generalizada de inflamação, e um dos marcadores laboratoriais principais é o aumento dos níveis de Dímero D, que está correlacionado com a gravidade dos sintomas clínicos.	Criticamente baixa
Maitra et al.^18^	Revisão sistemática e metanálise	Coorte e ECRs	19	Julho/2020	Incidência de TEV: - Incidência de TEV: 23% (10-36%; IC95%) - Incidência de EP: 12% (6-17%; IC95%) - Incidência de TVP: 15% (8-23%; IC95%)	Observou-se uma prevalência elevada de EP e TVP em indivíduos com covid-19, bem como uma considerável heterogeneidade nos eventos tromboembólicos em geral. Essa variação pode ser atribuída a fatores como a diversidade étnica dos pacientes, discrepâncias nos protocolos de triagem e profilaxia, agravamento da condição clínica e presença de comorbidades.	Criticamente baixa
Henrina et al.^19^	Revisão sistemática e metanálise	Coorte retrospectiva, prospectiva e transversal	45	Novembro/2020	Associação de TEV com fatores diversos: Há associações significativas entre o TEV e fatores como admissão em UTI (1,53-3,52; IC95%), sexo masculino (1,08-1,35; IC95%), aumento de Dímero D (2,74-6,25; IC95%), níveis de LDH (19,33-122,54; IC95%) e idade avançada (0,06-5,53; IC95%).	A ocorrência de TEV em pacientes afetados pela covid-19 está diretamente relacionada a uma maior probabilidade de necessidade de internação em UTI. Ainda, fatores como sexo masculino, idade avançada e elevados índices de contagem de leucócitos e Dímero D estão associados a um maior risco de desenvolvimento de TEV durante a infecção por covid-19.	Criticamente baixa
Suh et al.^20^	Revisão sistemática e metanálise	Coorte prospectiva e retrospectiva	27	Junho/2020	Incidência de EP e TVP: - Incidência de EP: 16,5% (IC95%: I2 = 0,93) - Incidência de TVP: 14,8% (IC95%: I2 = 0,94) EP foi encontrada com maior frequência em pacientes internados na UTI (24,7% [IC 95%]) *versus* aqueles não internados na UTI (10,5% [IC 95%]). A TVP esteve presente em 42,4% dos pacientes com EP.	Mais da metade dos pacientes que desenvolveram EP não apresentavam sinais de TVP concomitante. Os limiares de Dímero D utilizados como critério para descartar a ocorrência de EP, conforme definidos nas diretrizes prévias, parecem ser aplicáveis também aos pacientes diagnosticados com covid-19.	Baixa
Kollias et al.^21^	Revisão sistemática e metanálise	Observacionais	47	Setembro/2020	Prevalência de EP e TVP: - Prevalência de EP: 32% (25-40%; IC95%) - Prevalência de TVP: 27% (21-34%; IC95%)	Em pacientes hospitalizados com covid-19 e submetidos a triagem ou avaliação para TEV, a prevalência de TVP e EP é de aproximadamente 30% cada, mesmo com aplicação de medidas de tromboprofilaxia. Notavelmente, o risco de TEV parece ser elevado em comparação com pacientes que não apresentam covid-19 internados nas mesmas UTIs.	Criticamente baixa
Mazzaccaro et al.^22^	Revisão sistemática	Ensaios clínicos randomizados, estudos de coorte e séries de casos	69	Maio/2021	Prevalência de TEV: - Prevalência de TEV: 16,7% (5,8-30%), sendo maior em pacientes de UTI (60,8-85,4%). Idade avançada, Dímero D elevado e obesidade aumentaram as chances de desenvolver TEV. O sexo feminino pareceu ser protetor contra as chances de TEV.	Em indivíduos hospitalizados por covid-19, a ocorrência de TEV é frequentemente observada, com taxas de prevalência particularmente elevadas em pacientes internados em UTI. Fatores como idade avançada, obesidade e níveis elevados de Dímero D na admissão estão associados a um aumento nas probabilidades de TEV, enquanto o sexo feminino demonstrou ser protetor contra o desenvolvimento de TEV.	Criticamente baixa
Gabbai-Armelin et al.^23^	Revisão sistemática e metanálise	Coorte, transversal e caso-controle	20	Não informado	Prognósticos associados: Hipertensão e diabetes foram as comorbidades mais frequentemente associadas a eventos trombóticos.	Indivíduos com idade superior a 60 anos que apresentam histórico de hipertensão, diabetes e concentrações de Dímero D acima de 3,17 μg/mL podem ser identificados como fatores prognósticos associados ao desenvolvimento de TEV relacionado à covid-19.	Baixa
Roncon et al.^24^	Revisão sistemática e metanálise	Coorte retrospectiva e prospectiva	23	Agosto/2020	Incidência de EP: - Incidência EP: 14,7% (9,9-21,3%; IC95%; I2 = 95,0%) para pacientes em enfermaria e 23,4% (16,7-31,8%; IC95%; I2 = 88,7%) para pacientes em UTI.	A ocorrência de EP em ambiente hospitalar é mais frequente em pacientes com covid-19 que estão sob cuidados intensivos em comparação com aqueles internados em enfermarias gerais. Há ainda indícios de que os casos de EP podem estar subestimados.	Criticamente baixa
Xiong et al.^25^	Revisão sistemática e metanálise	Coorte retrospectiva, prospectiva e caso-controle	12	Julho/2020	Prevalência de trombose: - Prevalência de trombose: 22% (0,08-0,40; IC95%) em pacientes de enfermaria e 43% (0,29-0,65; IC 95%) em pacientes de UTI. Em comparação com os não trombóticos, os pacientes trombóticos apresentaram níveis mais elevados de Dímero D. Idade, contagem de plaquetas e sexo masculino tendem a ser riscos.	A prevalência de trombose em pacientes com covid-19 foi considerável, especialmente em UTI, apesar da administração de profilaxia farmacológica contra TEV. Portanto, é crucial dedicar especial atenção aos níveis elevados de Dímero D, LDH, leucócitos e à diminuição dos linfócitos em pacientes com covid-19.	Criticamente baixa
Longchamp et al.^26^	Revisão sistemática e metanálise	Coorte prospectiva e retrospectiva	33	Junho/2020	Incidência de TEV e EP: - Incidência de TEV: 9% (5-13%; IC95%) geral; 21% (14-28%; IC95%) para pacientes em UTI. - Incidência de EP: 8% (4-13%; IC95%) geral; 17% (11-25%; IC95%) para pacientes em UTI. - Incidência de TVP: 3% (1-5%; IC95%) geral; 8% (3-14%; IC95%) para pacientes em UTI.	O potencial de eventos tromboembólicos graves em pacientes hospitalizados com covid-19 é considerável. Contudo, sua incidência é sensível à gravidade da doença. Tais descobertas confirmam a necessidade de implementação de medidas tromboprofiláticas em todos os indivíduos hospitalizados por covid-19, além da realização de ensaios clínicos que avaliem distintos protocolos de tromboprofilaxia em subpopulações.	Baixa
Nopp et al.^27^	Revisão sistemática e metanálise	Coorte, transversal e caso-controle	86	Agosto/2020	Prevalência de TEV e EP: - Prevalência de TEV: 7,9% (5,1-11,2; IC95%) em pacientes fora da UTI e 22,7% (18,1-27,6; IC95%) para pacientes em UTI. - Prevalência de EP: 3,5% (2,2-5,1; IC95%) em pacientes fora da UTI e 13,7% (10,0-17,9; IC95%) para pacientes em UTI.	O risco de TEV é elevado em UTI. Entretanto, ele também se eleva em pacientes hospitalizados fora desse ambiente. Notavelmente, os indivíduos que manifestaram TEV demonstraram níveis significativamente mais elevados de Dímero D.	Baixa
Jenner et al.^28^	Revisão sistemática	Estudos observacionais	28	Novembro/2020	Taxa de complicações trombóticas: Complicações trombóticas ocorreram em 34% dos pacientes tratados na UTI, com TVP relatada em 16,1% e EP em 12,6%, apesar da tromboprofilaxia anticoagulante.	Mesmo com a administração de tromboprofilaxia anticoagulante, observa-se uma incidência considerável de complicações trombóticas em pacientes com covid-19 sob cuidados intensivos em UTI. Além disso, a implementação de triagem sistemática revela um número significativo de complicações trombóticas que poderiam passar despercebidas da suspeita clínica para iniciar a investigação. Recomenda-se fortemente o rastreamento sistemático de TEV.	Baixa
Wu et al.^29^	Revisão sistemática e metanálise	Estudos prospectivos e retrospectivos	39	Setembro/2020	Incidência de EP e TEV: Incidência de EP: 17% (13-21; IC95%) Incidência de TEV: 42% (25-60; IC95%) Pacientes do sexo masculino com covid-19 têm maior probabilidade de apresentar TEV.	O TEV é uma complicação frequentemente observada em pacientes gravemente afetados pela covid-19, estando fortemente relacionado a desfechos clínicos adversos.	Baixa
Birkeland et al.^30^	Revisão sistemática	Coorte retrospectiva prospectiva e transversal	14	Junho/2020	Incidência de TEV: Incidência de TEV: 26,9% (20,8-33,1; IC95%). As chances de TEV foram maiores na UTI (OR = 6,38; 3,67-11,11; IC95%).	Mesmo com uso de anticoagulação prévia, a incidência de TEV foi consideravelmente elevada.	Criticamente baixa
Mansory et al.^31^	Revisão sistemática e metanálise	Coorte retrospectiva, prospectiva, seção transversal, ECRs e série de casos	91	Dezembro/2020	Epidemiologia TEV: A frequência global de TEV em pacientes de UTI e não UTI foi de 24,1% (20,07-28,28; IC95%) e 7,7% (20,07-28,28; IC95%) respectivamente. EP ocorreu em 8,5% (6,91-10,20; IC95%) e TVP proximal ocorreu em 8,2% (6,67-9,87; IC95%) de todos os pacientes hospitalizados.	Há alta incidência de TEV em pacientes hospitalizados com covid-19, especialmente naqueles em UTI. No entanto, análises de sensibilidade indicam que as taxas previamente relatadas de TEV na covid-19 podem ter sido superestimadas.	Criticamente baixa
Porfidia et al.^32^	Revisão sistemática e metanálise	Coorte	30	Junho/2020	Incidência de TEV: - Incidência de TEV: 24% (95% IP, 5-66%) em pacientes de UTI e 9% (IP 95%, 0-94%) em pacientes de enfermarias. - Incidência de EP: 19% (95% IP, 6%-47%) em pacientes de UTI e 4% (IP 95%, 0-100%) em pacientes de enfermarias. -Incidência de TVP: 7% (95% IP, 0-69%) em pacientes de UTI e 7% (IC95%, 1-49%) em pacientes de enfermarias.	O TEV é uma complicação comum em pacientes internados por covid-19, sendo a EP uma apresentação recorrente. É crucial manter um nível de suspeita clínica elevado para identificar prontamente os casos.	Criticamente baixa
Zhang et al.^33^	Revisão sistemática e metanálise	Estudos prospectivos e retrospectivos	40	Agosto/2020	Prevalência de TEV: - Prevalência de TEV: 7% em pacientes fora da UTI (0,01-0,18; IC95%) e 31% em pacientes de UTI (0,22-0,42; IC95%). Pacientes de UTI também tiveram a maior prevalência de EP entre os grupos, 17% (0,12-0,23; IC95%), e a maior prevalência de TVP, 25% (0,14-0,37; IC95%).	O TEV é uma ocorrência frequente em pacientes hospitalizados com covid-19, sobretudo entre aqueles em UTI. A implementação de testes de triagem para EP e TVP pode melhorar significativamente as taxas de detecção em pacientes com covid-19 tanto na UTI quanto fora dela, em comparação com testes baseados exclusivamente em suspeita clínica.	Baixa

AMSTAR2 = Assessing the Methodological Quality of Systematic Reviews^12^; ECRs = ensaios clínicos randomizados; EP = embolia pulmonar; I2 = índice de heterogeneidade; IP = intervalo de predição; LDH = lactato desidrogenase; OR = *odds ratio* (razão de probabilidade); TEV = tromboembolismo venoso; TVP = tromboembolismo pulmonar; UTI = Unidade de Terapia Intensiva.

Em relação ao Dímero D, 12 estudos (70,58%) apontaram uma associação entre níveis elevados desse marcador e o risco de desenvolvimento de TEV em casos de covid-19, indicando que os pacientes com TEV apresentaram níveis mais altos^17,19-23,25,27,29,30^.

Ainda, cinco estudos (29,4%) indicaram que há maior risco de TEV como complicação da covid-19 para indivíduos do sexo masculino^18,19,22,25,29^.

Além disso, três estudos (17,64%) sugeriram que uma triagem sistemática com uso de exames de imagem para investigação de TEV é fortemente indicada, pois identifica complicações que podem passar despercebidas apenas com a avaliação clínica^28,32,34^.

Por fim, 15 estudos (88,23%) comprovaram que o risco de desenvolvimento de TEV é mais elevado para pacientes de UTI em relação aos pacientes hospitalizados fora da UTI, embora o risco também aumente para este grupo^17,19-22,24-32,34^.

## DISCUSSÃO

Entre os distúrbios de coagulação conhecidos, a resposta inflamatória da covid-19 manifesta principalmente o estado de hipercoagulabilidade, que tem como um de seus desfechos o TEV^5^. Sendo assim, este estudo concentrou-se nesse desfecho, estudando especialmente a EP e a TVP.

A covid-19, doença infecciosa causada pelo vírus SARS-CoV-2 e responsável pela pandemia de 2020 a 2023, gerou impacto mundial na saúde pública, e muitas questões sobre sua fisiopatologia e desdobramentos ainda não foram respondidas. Sabe-se que a doença causa, sobretudo, manifestações respiratórias e, de forma secundária, manifestações cardiovasculares, como a coagulopatia. Além disso, cada vez mais estudos têm provado sua forte relação com eventos tromboembólicos decorrentes de distúrbios de coagulação gerados pelos mecanismos da resposta inflamatória^34^.

A fisiopatologia da covid-19 envolve uma série de eventos complexos que ocorrem no organismo em resposta à infecção pelo vírus, como a resposta inflamatória, que inclui a liberação de citocinas pró-inflamatórias, como interleucina-6 (IL-6). Em alguns casos, essa resposta inflamatória pode ser exacerbada, o que leva a uma condição conhecida como “tempestade de citocinas”, que está associada a complicações graves^35^. Além disso, o vírus pode causar lesões às células endoteliais que revestem os vasos sanguíneos, o que contribui para a ativação do sistema de coagulação e pode resultar em trombose vascular. Esses eventos levam ao estado de hipercoagulabilidade, frequentemente observado em indivíduos acometidos pela doença^36^.

Nesta revisão, foram identificadas evidências de outras revisões que apontam o TEV como uma condição que ocorre em uma proporção significativa de pacientes hospitalizados com covid-19. A manifestação é claramente mais comum em pacientes internados em UTIs, o que se justifica por esse tipo de paciente atender aos três critérios da Tríade de Virchow, além de muitas vezes estar sujeito à imobilidade por períodos prolongados. Contudo, foi possível observar, por meio dos achados dos estudos selecionados, que pacientes fora da UTI também estão expostos aos riscos.

É válido mencionar que a obtenção de dados durante uma pandemia apresenta grandes desafios. No caso da pandemia de covid-19, devido à sua natureza emergente e de rápida propagação, houve a necessidade de gerar informações de forma rápida para guiar decisões clínicas, o que resultou em um ambiente de pesquisa científica acelerado e produtivo. Entretanto, devido às tantas limitações da época, como as restrições sanitárias, falta de recursos e diferentes condutas adotadas, a condução de estudos clínicos foi afetada, resultando em uma maior dependência de dados secundários. Como resultado, muitos dos dados disponíveis durante a pandemia foram obtidos de estudos observacionais e registros hospitalares, o que, apesar de valioso, pode introduzir vieses e limitar a interpretação dos resultados.

### Limitações do estudo

Os achados desta revisão corroboram a literatura atual em relação ao objeto de estudo. Entretanto, ainda há muitas lacunas no conhecimento acerca do tema e da própria fisiopatologia da covid-19. Destacamos que a adoção do atalho metodológico de limitar a busca a apenas duas bases de dados, embora não seja esperado, conforme descrito na Tabela 1, pode ter impactado na existência dessa lacuna. Ainda que existam muitos estudos primários acerca do tema, esses podem estar sendo pouco abordados em revisões de literatura.

Ressalta-se que nenhuma das revisões incluídas e avaliadas apresentou uma qualidade metodológica alta, conforme verificado com a ferramenta AMSTAR2^13^. Dessa forma, os resultados e considerações levantados devem ser usados com atenção, uma vez que a qualidade metodológica baixa ou criticamente baixa das revisões aponta para possíveis vieses na descrição dos achados.

Esta revisão não avaliou a mortalidade e nem os possíveis tratamentos das condições abordadas, focando exclusivamente nos resultados intra-hospitalares. Futuras pesquisas poderão investigar os efeitos a longo prazo e o manejo da covid-19 associada ao TEV. Outra limitação relevante deste estudo é que a maioria dos artigos incluídos são de 2020 e 2021. Lembramos que o critério de inclusão desta revisão rápida foi incluir publicações de revisões sistemáticas, o que, por um lado, não indica a falta de estudos primários realizados sobre o assunto, mas instiga a necessidade de se realizar revisões de literatura sobre o tema, a fim de termos mais sínteses de evidências científicas disponíveis.

### Contribuições para a prática clínica

A partir dos achados, sugere-se realizar trabalhos de revisão que incluam um comparador, como “paciente de UTI sem covid-19 *versus* paciente de UTI com covid-19”, para refinar os dados. Sugere-se também a elaboração de um instrumento para avaliação de risco de TEV direcionada ao paciente com covid-19 em UTI.

A continuidade na investigação do tema e a publicação de achados relacionados são essenciais para que a temática continue contribuindo para a sociedade e para os profissionais da saúde interessados no tema.

## CONCLUSÃO

A partir das evidências científicas disponíveis, considerando-se a baixa disponibilidade de estudos secundários e sua baixa qualidade metodológica, identificamos e sintetizamos três pontos principais quanto a associação de quadros de covid-19 e desenvolvimento de TEV: primeiro, que a incidência de eventos tromboembólicos (TVP e/ou EP) como uma complicação associada a quadros de covid-19 é alta em adultos hospitalizados, uma vez que esse quadro desencadeia uma resposta inflamatória sistêmica no corpo, que pode muitas vezes acarretar distúrbios de coagulação, como hipercoagulabilidade e lesão endotelial, especialmente em pacientes de UTIs, que estão expostos a mais fatores de risco, como imobilidade e lesão endotelial associada a dispositivos vasculares. Segundo, que níveis de Dímero D elevados são comumente encontrados em pacientes graves, sendo esse um indicador de mau prognóstico por estar associado a maior risco de complicações tromboembólicas. Terceiro, que é necessária maior atenção ao rastreio de TEV em pacientes hospitalizados com covid-19 utilizando marcadores laboratoriais como o Dímero D e exames de imagem, e, se adequado, profilaxia antitrombótica conforme as diretrizes, a fim de prevenir complicações.

## Material Suplementar

Este artigo acompanha material suplementar.

Tabela suplementar 1. Base de dados, estratégia de busca e número de publicações encontradas.

Tabela suplementar 2. Lista de artigos excluídos durante a seleção de dados e sua respectiva justificativa.

Este material está disponível como parte da versão online do artigo na página https://doi.org/10.1590/1677-5449.202400731
